# Supplementary material for: Modeling glioblastoma heterogeneity as a dynamic network of cell states
Source: Mol Syst Biol. 2021 Sep 16;17(9):e10105. doi: 10.15252/msb.202010105 (PMC8444284; doi:10.15252/msb.202010105)
Supplement: Supplementary file 5 — Source Data for Figure 3 [file MSB-17-e10105-s001.zip › Figure3A_sourcedata/GSEA_3065/hallmarks_state5.GseaPreranked.1623416302988/HALLMARK_G2M_CHECKPOINT.html]

Details for gene set HALLMARK\_G2M\_CHECKPOINT[GSEA]

|  || Dataset | state5 |
| Phenotype | NoPhenotypeAvailable |
| Upregulated in class | na\_neg |
| GeneSet | HALLMARK\_G2M\_CHECKPOINT |
| Enrichment Score (ES) | -0.37565467 |
| Normalized Enrichment Score (NES) | -1.3696448 |
| Nominal p-value | 0.013745705 |
| FDR q-value | 0.123096965 |
| FWER p-Value | 0.834 |
Table: GSEA Results Summary

  

Fig 1: Enrichment plot: HALLMARK\_G2M\_CHECKPOINT      
 Profile of the Running ES Score & Positions of GeneSet Members on the Rank Ordered List

  

| PROBE | GENE SYMBOL | GENE\_TITLE | RANK IN GENE LIST | RANK METRIC SCORE | RUNNING ES | CORE ENRICHMENT || 1 | SQLE |  |  | 39 | 0.705 | 0.0228 | No |
| 2 | LBR |  |  | 198 | 0.440 | 0.0219 | No |
| 3 | SLC12A2 |  |  | 469 | 0.320 | 0.0036 | No |
| 4 | MTF2 |  |  | 677 | 0.269 | -0.0096 | No |
| 5 | NOTCH2 |  |  | 756 | 0.249 | -0.0088 | No |
| 6 | ORC6 |  |  | 806 | 0.240 | -0.0051 | No |
| 7 | XPO1 |  |  | 933 | 0.220 | -0.0109 | No |
| 8 | SLC38A1 |  |  | 992 | 0.212 | -0.0093 | No |
| 9 | NASP |  |  | 1225 | 0.182 | -0.0286 | No |
| 10 | SRSF10 |  |  | 1270 | 0.176 | -0.0268 | No |
| 11 | ILF3 |  |  | 1314 | 0.170 | -0.0252 | No |
| 12 | POLQ |  |  | 1338 | 0.168 | -0.0213 | No |
| 13 | NUMA1 |  |  | 1360 | 0.165 | -0.0173 | No |
| 14 | CENPF |  |  | 1531 | 0.149 | -0.0309 | No |
| 15 | CCNT1 |  |  | 1645 | 0.138 | -0.0384 | No |
| 16 | RBL1 |  |  | 1669 | 0.136 | -0.0357 | No |
| 17 | MEIS1 |  |  | 1728 | 0.131 | -0.0373 | No |
| 18 | BRCA2 |  |  | 1781 | 0.128 | -0.0382 | No |
| 19 | PRIM2 |  |  | 1881 | 0.120 | -0.0448 | No |
| 20 | WRN |  |  | 1933 | 0.117 | -0.0461 | No |
| 21 | KIF20B |  |  | 1962 | 0.114 | -0.0449 | No |
| 22 | PDS5B |  |  | 1989 | 0.112 | -0.0435 | No |
| 23 | TNPO2 |  |  | 2182 | 0.100 | -0.0615 | No |
| 24 | HNRNPU |  |  | 2204 | 0.098 | -0.0601 | No |
| 25 | MEIS2 |  |  | 2207 | 0.098 | -0.0565 | No |
| 26 | TRA2B |  |  | 2285 | 0.093 | -0.0617 | No |
| 27 | SRSF1 |  |  | 2334 | 0.090 | -0.0637 | No |
| 28 | POLE |  |  | 2438 | 0.083 | -0.0722 | No |
| 29 | SLC7A1 |  |  | 2527 | 0.077 | -0.0792 | No |
| 30 | SFPQ |  |  | 2548 | 0.077 | -0.0785 | No |
| 31 | TENT4A |  |  | 2603 | 0.073 | -0.0818 | No |
| 32 | ARID4A |  |  | 2629 | 0.072 | -0.0819 | No |
| 33 | SLC7A5 |  |  | 2658 | 0.071 | -0.0823 | No |
| 34 | RASAL2 |  |  | 2733 | 0.066 | -0.0882 | No |
| 35 | NSD2 |  |  | 2788 | 0.063 | -0.0919 | No |
| 36 | CCNF |  |  | 2816 | 0.062 | -0.0925 | No |
| 37 | AURKB |  |  | 2853 | 0.060 | -0.0943 | No |
| 38 | CHEK1 |  |  | 2877 | 0.059 | -0.0947 | No |
| 39 | LIG3 |  |  | 2883 | 0.058 | -0.0930 | No |
| 40 | SRSF2 |  |  | 2908 | 0.057 | -0.0935 | No |
| 41 | DMD |  |  | 2914 | 0.057 | -0.0918 | No |
| 42 | STAG1 |  |  | 2972 | 0.054 | -0.0963 | No |
| 43 | CASP8AP2 |  |  | 3046 | 0.050 | -0.1026 | No |
| 44 | E2F3 |  |  | 3048 | 0.050 | -0.1008 | No |
| 45 | PRPF4B |  |  | 3071 | 0.048 | -0.1014 | No |
| 46 | NUP98 |  |  | 3316 | 0.035 | -0.1278 | No |
| 47 | SMC2 |  |  | 3374 | 0.032 | -0.1330 | No |
| 48 | CDC6 |  |  | 3392 | 0.032 | -0.1337 | No |
| 49 | E2F4 |  |  | 3422 | 0.031 | -0.1358 | No |
| 50 | NUP50 |  |  | 3457 | 0.029 | -0.1386 | No |
| 51 | TGFB1 |  |  | 3503 | 0.027 | -0.1426 | No |
| 52 | SS18 |  |  | 3535 | 0.026 | -0.1451 | No |
| 53 | MAPK14 |  |  | 3545 | 0.025 | -0.1452 | No |
| 54 | EWSR1 |  |  | 3617 | 0.023 | -0.1524 | No |
| 55 | ORC5 |  |  | 3620 | 0.023 | -0.1517 | No |
| 56 | SMC1A |  |  | 3696 | 0.020 | -0.1595 | No |
| 57 | CDC7 |  |  | 3855 | 0.014 | -0.1769 | No |
| 58 | SMAD3 |  |  | 3915 | 0.011 | -0.1832 | No |
| 59 | ATRX |  |  | 3933 | 0.010 | -0.1847 | No |
| 60 | KNL1 |  |  | 3943 | 0.009 | -0.1854 | No |
| 61 | PML |  |  | 3947 | 0.009 | -0.1854 | No |
| 62 | KIF15 |  |  | 3950 | 0.009 | -0.1852 | No |
| 63 | RBM14 |  |  | 3995 | 0.007 | -0.1899 | No |
| 64 | TLE3 |  |  | 4004 | 0.007 | -0.1906 | No |
| 65 | NDC80 |  |  | 4034 | 0.006 | -0.1937 | No |
| 66 | PRC1 |  |  | 4241 | -0.003 | -0.2169 | No |
| 67 | MNAT1 |  |  | 4417 | -0.011 | -0.2364 | No |
| 68 | TACC3 |  |  | 4450 | -0.012 | -0.2396 | No |
| 69 | CDC45 |  |  | 4563 | -0.017 | -0.2516 | No |
| 70 | KIF2C |  |  | 4621 | -0.019 | -0.2574 | No |
| 71 | CUL5 |  |  | 4627 | -0.019 | -0.2572 | No |
| 72 | FOXN3 |  |  | 4639 | -0.020 | -0.2577 | No |
| 73 | MYC |  |  | 4656 | -0.020 | -0.2587 | No |
| 74 | PURA |  |  | 4668 | -0.021 | -0.2592 | No |
| 75 | CDK1 |  |  | 4766 | -0.025 | -0.2692 | No |
| 76 | BARD1 |  |  | 4776 | -0.025 | -0.2693 | No |
| 77 | CENPE |  |  | 4787 | -0.025 | -0.2695 | No |
| 78 | MCM2 |  |  | 4824 | -0.027 | -0.2725 | No |
| 79 | HMMR |  |  | 4826 | -0.027 | -0.2716 | No |
| 80 | EZH2 |  |  | 4858 | -0.028 | -0.2741 | No |
| 81 | TROAP |  |  | 4869 | -0.028 | -0.2741 | No |
| 82 | MAP3K20 |  |  | 4920 | -0.030 | -0.2786 | No |
| 83 | DDX39A |  |  | 4948 | -0.031 | -0.2805 | No |
| 84 | GINS2 |  |  | 5024 | -0.034 | -0.2877 | No |
| 85 | HIRA |  |  | 5060 | -0.035 | -0.2903 | No |
| 86 | KATNA1 |  |  | 5142 | -0.038 | -0.2981 | No |
| 87 | PRMT5 |  |  | 5155 | -0.039 | -0.2979 | No |
| 88 | UPF1 |  |  | 5188 | -0.040 | -0.3000 | No |
| 89 | BUB1 |  |  | 5195 | -0.040 | -0.2992 | No |
| 90 | ODF2 |  |  | 5219 | -0.041 | -0.3002 | No |
| 91 | POLA2 |  |  | 5229 | -0.042 | -0.2996 | No |
| 92 | YTHDC1 |  |  | 5276 | -0.043 | -0.3032 | No |
| 93 | UCK2 |  |  | 5343 | -0.046 | -0.3089 | No |
| 94 | CCNB2 |  |  | 5382 | -0.048 | -0.3113 | No |
| 95 | NUSAP1 |  |  | 5389 | -0.048 | -0.3102 | No |
| 96 | NEK2 |  |  | 5442 | -0.050 | -0.3141 | No |
| 97 | CDC25A |  |  | 5443 | -0.050 | -0.3122 | No |
| 98 | CTCF |  |  | 5558 | -0.055 | -0.3230 | No |
| 99 | BUB3 |  |  | 5596 | -0.057 | -0.3250 | No |
| 100 | TTK |  |  | 5604 | -0.058 | -0.3236 | No |
| 101 | CCNA2 |  |  | 5671 | -0.061 | -0.3287 | No |
| 102 | TOP2A |  |  | 5722 | -0.063 | -0.3320 | No |
| 103 | KIF22 |  |  | 5758 | -0.064 | -0.3335 | No |
| 104 | KIF11 |  |  | 5795 | -0.065 | -0.3351 | No |
| 105 | KPNA2 |  |  | 5811 | -0.066 | -0.3342 | No |
| 106 | DR1 |  |  | 5872 | -0.068 | -0.3384 | No |
| 107 | RACGAP1 |  |  | 5902 | -0.070 | -0.3390 | No |
| 108 | ABL1 |  |  | 5987 | -0.073 | -0.3457 | No |
| 109 | CDKN1B |  |  | 5996 | -0.074 | -0.3438 | No |
| 110 | DKC1 |  |  | 6027 | -0.076 | -0.3443 | No |
| 111 | G3BP1 |  |  | 6152 | -0.082 | -0.3552 | No |
| 112 | CHAF1A |  |  | 6157 | -0.082 | -0.3524 | No |
| 113 | CUL4A |  |  | 6188 | -0.084 | -0.3526 | No |
| 114 | MKI67 |  |  | 6211 | -0.086 | -0.3518 | No |
| 115 | CUL1 |  |  | 6216 | -0.086 | -0.3489 | No |
| 116 | HUS1 |  |  | 6314 | -0.091 | -0.3564 | No |
| 117 | CENPA |  |  | 6374 | -0.094 | -0.3595 | No |
| 118 | SMC4 |  |  | 6517 | -0.102 | -0.3717 | Yes |
| 119 | CHMP1A |  |  | 6523 | -0.103 | -0.3683 | Yes |
| 120 | E2F1 |  |  | 6528 | -0.103 | -0.3648 | Yes |
| 121 | KIF4A |  |  | 6529 | -0.103 | -0.3608 | Yes |
| 122 | ATF5 |  |  | 6540 | -0.104 | -0.3579 | Yes |
| 123 | SMARCC1 |  |  | 6547 | -0.105 | -0.3545 | Yes |
| 124 | MCM5 |  |  | 6553 | -0.105 | -0.3510 | Yes |
| 125 | DBF4 |  |  | 6572 | -0.106 | -0.3489 | Yes |
| 126 | PLK1 |  |  | 6581 | -0.107 | -0.3457 | Yes |
| 127 | MAD2L1 |  |  | 6600 | -0.108 | -0.3436 | Yes |
| 128 | LMNB1 |  |  | 6638 | -0.110 | -0.3435 | Yes |
| 129 | PAFAH1B1 |  |  | 6702 | -0.114 | -0.3463 | Yes |
| 130 | RPA2 |  |  | 6705 | -0.114 | -0.3421 | Yes |
| 131 | KIF23 |  |  | 6764 | -0.118 | -0.3442 | Yes |
| 132 | FBXO5 |  |  | 6775 | -0.119 | -0.3407 | Yes |
| 133 | TOP1 |  |  | 6911 | -0.130 | -0.3510 | Yes |
| 134 | CDC20 |  |  | 6985 | -0.136 | -0.3541 | Yes |
| 135 | AURKA |  |  | 6990 | -0.136 | -0.3493 | Yes |
| 136 | HIF1A |  |  | 6996 | -0.137 | -0.3445 | Yes |
| 137 | PBK |  |  | 7010 | -0.138 | -0.3407 | Yes |
| 138 | CDC27 |  |  | 7083 | -0.144 | -0.3433 | Yes |
| 139 | CDC25B |  |  | 7102 | -0.146 | -0.3397 | Yes |
| 140 | CUL3 |  |  | 7103 | -0.146 | -0.3341 | Yes |
| 141 | KPNB1 |  |  | 7144 | -0.150 | -0.3329 | Yes |
| 142 | TFDP1 |  |  | 7187 | -0.154 | -0.3317 | Yes |
| 143 | RAD23B |  |  | 7194 | -0.155 | -0.3264 | Yes |
| 144 | TMPO |  |  | 7296 | -0.167 | -0.3315 | Yes |
| 145 | SYNCRIP |  |  | 7350 | -0.175 | -0.3307 | Yes |
| 146 | MCM6 |  |  | 7375 | -0.178 | -0.3266 | Yes |
| 147 | TPX2 |  |  | 7549 | -0.197 | -0.3386 | Yes |
| 148 | MCM3 |  |  | 7552 | -0.198 | -0.3312 | Yes |
| 149 | BIRC5 |  |  | 7596 | -0.204 | -0.3282 | Yes |
| 150 | NOLC1 |  |  | 7699 | -0.218 | -0.3314 | Yes |
| 151 | RAD21 |  |  | 7785 | -0.231 | -0.3321 | Yes |
| 152 | KMT5A |  |  | 7815 | -0.237 | -0.3262 | Yes |
| 153 | SAP30 |  |  | 7936 | -0.263 | -0.3297 | Yes |
| 154 | AMD1 |  |  | 7944 | -0.264 | -0.3203 | Yes |
| 155 | CKS2 |  |  | 7972 | -0.270 | -0.3129 | Yes |
| 156 | MYBL2 |  |  | 8041 | -0.283 | -0.3097 | Yes |
| 157 | DTYMK |  |  | 8075 | -0.292 | -0.3022 | Yes |
| 158 | CDKN3 |  |  | 8079 | -0.293 | -0.2912 | Yes |
| 159 | CDKN2C |  |  | 8091 | -0.295 | -0.2811 | Yes |
| 160 | CBX1 |  |  | 8222 | -0.331 | -0.2830 | Yes |
| 161 | KIF5B |  |  | 8235 | -0.334 | -0.2715 | Yes |
| 162 | CKS1B |  |  | 8241 | -0.336 | -0.2591 | Yes |
| 163 | UBE2C |  |  | 8278 | -0.348 | -0.2497 | Yes |
| 164 | GSPT1 |  |  | 8299 | -0.355 | -0.2383 | Yes |
| 165 | HSPA8 |  |  | 8339 | -0.366 | -0.2286 | Yes |
| 166 | CCND1 |  |  | 8346 | -0.368 | -0.2151 | Yes |
| 167 | HMGB3 |  |  | 8427 | -0.399 | -0.2087 | Yes |
| 168 | HNRNPD |  |  | 8436 | -0.402 | -0.1941 | Yes |
| 169 | PTTG1 |  |  | 8446 | -0.406 | -0.1795 | Yes |
| 170 | NCL |  |  | 8496 | -0.433 | -0.1683 | Yes |
| 171 | SNRPD1 |  |  | 8537 | -0.451 | -0.1554 | Yes |
| 172 | HMGN2 |  |  | 8608 | -0.496 | -0.1442 | Yes |
| 173 | CDK4 |  |  | 8625 | -0.510 | -0.1264 | Yes |
| 174 | JPT1 |  |  | 8647 | -0.523 | -0.1086 | Yes |
| 175 | MARCKS |  |  | 8667 | -0.537 | -0.0900 | Yes |
| 176 | HMGA1 |  |  | 8675 | -0.543 | -0.0698 | Yes |
| 177 | MT2A |  |  | 8740 | -0.598 | -0.0540 | Yes |
| 178 | STMN1 |  |  | 8783 | -0.638 | -0.0341 | Yes |
| 179 | ODC1 |  |  | 8839 | -0.697 | -0.0135 | Yes |
| 180 | UBE2S |  |  | 8901 | -0.772 | 0.0094 | Yes |
Table: GSEA details [plain text format]

  

Fig 2: HALLMARK\_G2M\_CHECKPOINT: Random ES distribution      
 Gene set null distribution of ES for **HALLMARK\_G2M\_CHECKPOINT**
